# Characteristics of *Chlamydia pneumoniae* infection in 141 children with respiratory tract infection and risk factors for severe pneumonia

**DOI:** 10.3389/fped.2026.1879436

**Published:** 2026-08-28

**Authors:** Shu Li, Lina Chen, Hanmin Liu

**Affiliations:** Division of Pediatric Pulmonology and Immunology, Key Laboratory of Birth Defects and Related Diseases of Women and Children (Sichuan University), Ministry of Education, West China Second University Hospital, Sichuan University, Chengdu, China

**Keywords:** children, Chlamydia pneumoniae, pneumonia, risk factors, severe pneumonia

## Abstract

**Purpose:**

Literature on *Chlamydia pneumoniae* infection in children is far less abundant than that on *Mycoplasma pneumoniae* infection. This study aims to describe the characteristics of Chlamydia pneumoniae (C. pneumoniae) respiratory tract infection in children and to identify the risk factors for cases with severe pneumonia.

**Methods:**

Data of 141 children with C. pneumoniae respiratory tract infection were collected from a university tertiary hospital from June 2019 to April 2025. Patients were divided into 3 groups: non-pneumonia (*N* = 64), pneumonia (*N* = 47) and severe-pneumonia groups (*N* = 30). Differences among groups were compared of demographic, clinical, biochemical and radiological data. Risk factors for severe C. pneumoniae pneumonia (CPP) were analyzed using multivariate binary logistic regression analysis.

**Results:**

Data of 141 children (61 males and 80 females) with a median age of 8.0 years (interquartile range 6.7–9.9) were collected. C. pneumoniae respiratory tract infection peaked in winter (58/141, 41.1%) in school-aged children (≥7.0 years old, 99/141, 70.2%) with major clinical manifestations being cough (135/141, 95.7%) and fever (69/141, 48.9%). Radiological manifestations included patchy opacities and consolidation in the pneumonia group and pleural effusion, lobar pulmonary consolidation, pulmonary atelectasis and “white lung” like changes (diffuse bilateral pulmonary opacification) in the severe-pneumonia group. Median time of hospital stay was 8.0 days in pneumonia group and 15.0 days in severe-pneumonia group, respectively. Multivariate binary logistic regression identified elevated wheezing (OR = 15.468, 95% CI: 2.845–84.101, *P* = 0.002) and coinfection (OR = 8.253, 95% CI:1.763–38.630, *P* = 0.007) as factors for severe CPP.

**Conclusions:**

Respiratory tract infection of C. pneumoniae in children mainly occurs in winter in school-aged children with cough and fever being the major clinical manifestations. Wheezing and coinfection are associated with severe CPP in children, suggesting potential roles in risk stratification.

## Introduction

Chlamydia is an obligate intracellular prokaryotic organism that cannot synthesize ATP and does not respond to β-lactam antibiotics without cell wall (1). Since 1957, it has been classified as a gram-negative bacterium. Currently, 11 species of Chlamydia are known to be pathogenic to humans or animals: Chlamydia trachomatis, Chlamydia pneumoniae, Chlamydia psittaci, etc. Among them, Chlamydia trachomatis and Chlamydia pneumoniae are the main pathogens causing human diseases. Most people experience multiple infections of Chlamydia in their lifetime (2, 3).

With only 1 serotype, Chlamydia pneumoniae infection may be asymptomatic or can cause various respiratory diseases in humans, such as pharyngitis and atypical pneumonia. C. pneumoniae pneumonia accounts for about 10% of community-acquired pneumonia in all populations (4). C. pneumoniae infection also relates to chronic diseases such as asthma, atherosclerosis, and arthritis (5).

Approximately 10%–20% of children have C. pneumoniae antibodies. C. pneumoniae infection mainly occurs in school-aged children and adolescents. In Matheus's study including 416 children, C. pneumoniae was detected in 8.5% of children with upper respiratory tract infection and 3.3% of children with pneumonia (6). In the past two decades, studies have declined on C. pneumoniae (7, 8) due to its similarity to Mycoplasma pneumoniae in both clinical manifestations and therapeutic management. However, during the post-COVID-19 period, the detection rate of atypical pathogens rebounded in many regions worldwide including *Chlamydia pneumoniae (*9–12). Thus aroused our interest in investigating C. pneumonia. This study reported on the clinical characteristics of pediatric C. pneumoniae respiratory tract infection and risk factors for severe C. pneumoniae pneumonia.

### What is known—what is New

Chlamydia pneumoniae plays a part in pediatric respiratory tract infection and may be associated with certain chronic and immunological disorders (e.g., asthma, atherosclerosis, and arthritis). This study was to enhance our understanding of the clinical characteristics of C. pneumoniae respiratory tract infection in children and the risk factors for severe CPP, which is an important etiological agent of pediatric Community-acquired pneumonia.

## Subjects and methods

### Case selection and data collection

Inclusion criteria: (1) Age: 18 years or younger; (2) Patients visiting the outpatient/ emergency/ inpatient department of our university tertiary hospital from June 2019 to April 2025. (3) Having symptoms of respiratory tract infections such as fever, cough, wheeze and dyspnea; (2) Positive for serum CP-IgM or nucleic acid detection in respiratory tract secretion specimens (13) (see the detection method for details). The diagnostic criteria for different kinds of respiratory tract infections (acute upper respiratory tract infection, acute bronchitis, pneumonia, severe pneumonia) were based on the literature (14–16). A diagnosis of severe pneumonia was established if any of the following criteria was met: (1) poor general condition (2) refusal to feed of signs of dehydration (3) altered mental status (4) significantly elevated respiratory rate (defined as >70 breaths/min in infants or >50 breaths/min in older children) (5) cyanosis (6) dyspnea (grunting, nasal flaring or retractions) (7) multi-lobar involvement or infiltrates affecting ≥2/3 of the unilateral lung radiographically (8) pleural effusion (9) pulse oxygen saturation (SpO_2_) ≤ 92% (10) extrapulmonary complications (e.g., meningitis, osteomyelitis or sepsis) (14).

Exclusion criteria: (1) History of inborn errors of immunity and malignancy; (2) Pneumonia occurring 48 h after hospitalization; (3) Infants under 6 months of age who were positive for Chlamydia nucleic acid detection by method of multiplex pathogen nucleic acid detection (To exclude Chlamydia trachomatis infection); (4) Extrapulmonary infections such as intracranial, urinary tract or digestive tract infections.

#### Ethical Approval

This study was a clinical retrospective study and received an exemption from informed consent. (This study is assessed and approved by the Medical Ethics Committee of West China Second University Hospital, Sichuan University, Ethics No.: 2022074).

### Detection methods

The following methods were used for C. pneumoniae detection in this experiment: 1. Serum C. pneumoniae antibody determination: The enzyme-linked immunosorbent assay (ELISA) was used to determine the C. pneumoniae IgM/IgG antibodies in the samples. When the S/CO value (sample value/cut-off value) >1.1, it was judged as positive (Chlamydia pneumoniae IgM and IgG antibody detection kits, Zhuhai Livzon Reagent Co., Ltd.); 2. Fluorescent PCR detection of C. pneumoniae DNA in respiratory tract specimens (pharyngeal swabs, nasopharyngeal swabs, nasopharyngeal aspirates, bronchoalveolar lavage fluids). The positive standard was a Ct value of ≤38, and the detection sensitivity was 5.0 × 10^2^ copies/mL (Chlamydia pneumoniae nucleic acid detection kit, Xiamen Anpuli Bio—Engineering Co., Ltd.).

Detection methods for coinfection: 1. The colloidal gold method and the agglutination assay were used for the detection of Mycoplasma pneumoniae IgM antibodies and the total antibodies (Mycoplasma pneumoniae antibody detection kits, Zhuhai Livzon Reagent Co., Ltd.); 2. Seven-in-one detection of respiratory viruses (direct immunofluorescence antigen detection of nasopharyngeal swabs); 3. Multiplex nucleic acid detection of respiratory pathogens: Advanced Fragment Analysis, AFA. The detection of Chlamydia nucleic acid was both for Chlamydia pneumoniae or Chlamydia trachomatis.

### Statistical analysis

SPSS statistics 21.0 software was used for data processing and analysis. Quantitative data was expressed as $\bar{x}\pm s$ when normally distributed and as median and the interquartile range when not normally distributed. Considering data integrity, intergroup comparisons of clinical data were performed between the pneumonia and severe-pneumonia group using the Kruskal–Wallis *H*-test when data was not normally distributed. To go further, Mann–Whitney *U*-test was used for pairwise comparison between groups. The *χ*^2^-test was used for intergroup comparisons of categorical variables. Backward LR multivariate binary logistic regression was used for risk factor analysis of severe C. pneumoniae pneumonia. A two-sided *P* < 0.05 was considered statistically significant.

## Results

### Age-, sex- and seasonal pattern analysis

One hundred and forty-eight children with Chlamydia pneumoniae respiratory tract infections were collected from the outpatient/emergency/inpatient department of our hospital from June 2019 to April 2025. 7 cases meeting the exclusion criteria were exclude (2 acute leukemia, 3 under 6 months old who were positive for Chlamydia nucleic acid detection and 2 with abdominal or urinary infection). Data of 141 cases (including gender, age, clinical manifestation, laboratory and radiological results, underlying diseases, complications and treatments) were collected and analyzed. Children were divided into non-pneumonia (*N* = 64), pneumonia (*N* = 47) and severe-pneumonia groups (*N* = 30) according to their disease diagnosis. The non-pneumonia group includes 1 case with acute upper respiratory tract infection and 63 cases with acute bronchitis.

Among the 141 children, 137 cases were positive for C. pneumoniae-IgM antibody and 6 cases were positive for C. pneumoniae DNA in pharyngeal swab assays. The median duration from onset of respiratory symptoms to positive antibody test of C. pneumoniae was 10.0 days (maximum 20.0 days and minimum 6.0 days). Notably, in the 141 children with C. pneumoniae infection, 60 cases (60/141, 42.5%) were both positive for M. pneumoniae with positive IgM-antibody or with a total antibody titer ≥1:1,280.

The 141 children included 61 males and 80 females with the youngest being one month old and the oldest 15.1 years old (median age 8.0 years, interquartile range 6.7–9.9). The age and sex distribution were shown in Table 1. There was a significant difference in age distribution among the three groups (*χ*^2^ = 24.093, *P* = 0.000). *Post-hoc* pairwise comparisons with Bonferroni correction revealed that patients in severe-pneumonia group were significantly younger than those in pneumonia group (Z = −3.312, *P* = 0.003) and non-pneumonia group (Z = −4.685, *P* = 0.000). There was no statistically significant difference in gender distribution among the three groups (*χ*^2^ = 0.739, *P* = 0.691). In terms of seasonal distribution, C. pneumoniae infection mainly occurred in winter (58/141, 41.1%), with the rest seasons follows: autumn (31/141, 21.9%), summer (27/141, 19.1%) and spring (25/141, 17.7%).

**Table 1:** Demographic characteristics of the 141 children with Chlamydia pneumoniae respiratory tract infection.

Demographic data/group	Non-pneumonia group^a^	Pneumonia group^b^	Severe-pneumonia group^c^	Statistic	*P*-value
	(*N* = 64)	(*N* = 47)	(*N* = 30)		
Age (IQR*)-yr	8.8 (7.5, 10.3)	7.8 (6.8, 9.5)	5.2 (0.7, 7.4)	*χ*^2^ = 24.093	0.000
	a vs. b**			Z = −2.099	0.108
	b vs. c**			Z = −3.312	0.003
	a vs. c**			Z = −4.685	0.000
Female-no. (%)	37 (57.8)	28 (59.6)	15 (50.0)	χ^2^ = 0.739	0.691
Male-no. (%)	27 (42.2)	19 (40.4)	15 (50.0)		

* IQR: interquartile range.

** a: non-pneumonia group b: pneumonia group c: severe-pneumonia group.

### Major clinical manifestations

#### Clinical manifestations

Among the 141 children 135 had various degree of cough (127 productive cough and 8 children dry cough). 69 children had fever (69/141, 48.9%), among which 42 cases high fever above 39℃ (42/69, 60.8%) and 16 cases moderate fever (16/69, 23.1%). 18 children had wheezing and 16 had shortness of breaths. 11 children had headache or dizziness, 8 sore throats or pharyngeal discomfort. 4 children had abdominal pain and 4 chest pain. 40 cases (40/141, 28.3%) were positive for pulmonary physical examination. Details of the pneumonia (*N* = 47) and severe-pneumonia group (*N* = 30) were compared in Table 2 including clinical manifestations, biochemical parameters, length of hospital stays and complications.

**Table 2:** Clinical characteristics of the 77 children with Chlamydia pneumoniae pneumonia.

Clinical parameters/group	Pneumonia group	Severe-pneumonia group	Statistic	*P*-value
	(*N* = 47)	(*N* = 30)		
Fever	33 (70.2%)	24 (80.0%)	χ^2^ = −0.297	0.767
Productive Cough	25 (53.1%)	22 (73.3%)	χ^2^ = −0.316	0.752
Wheezing	2 (4.2%)	16 (53.3%)	χ^2^ = −4.287	0.000
Rhinorrhea	15 (31.9%)	8 (26.6%)	χ^2^ = −0.557	0.578
Nasal Obstruction	3 (6.3%)	5 (16.6%)	χ^2^ = −1.068	0.286
Dry Rales	3 (6.3%)	15 (50.0%)	χ^2^=−3.966	0.000
Moist Rales	22 (46.8%)	23 (76.6%)	χ^2^ = −2.849	0.004
Peripheral Blood WBC (×10^9^/L)	8.5 (5.7,12.4)	9.7 (5.7,15.3)	Z = 0.50	0.617
Neutrophil Count (×10^9^/L)	4.24 (3.37, 7.78)	3.44 (2.68, 8.27)	Z = 0.93	0.347
Neutrophil Percentage (%)	60.9 (52.3,70.2)	67.6 (48.0,75.6)	Z = 0.90	0.366
NLR^a^	1.91 (1.31,2.93)	2.29 (0.75,4.35)	Z = 0.07	0.941
Hemoglobin (g/L)	118 (114,129)	118 (96.5,131)	Z = 0.16	0.869
Platelets (×10^9^/L)	276 (195,434)	346 (233,444)	Z = 0.91	0.362
C-reactive Protein (mg/L)	12.6 (2.0,30.3)	12.1 (1.9,39.7)	Z = 0.14	0.888
ALT^a^ (U/L)	18 (11.5,27.5)	34 (16,49)	Z = 2.08	0.037
LDH^a^ (U/L)	294 (193,580)	380 (283,866)	Z = 1.65	0.098
Hospital stays (day)	8.0 (6.0,13.5)	15.0 (9.5,23.0)	Z = 0.19	0.056

a NLR: the ratio of neutrophils to lymphocytes; ALT: alanine aminotransferase; LDH: lactate dehydrogenase.

In pneumonia group (*N* = 47) 15 cases were admitted to the inpatient department and the rest 32 cases received treatment at the outpatient/emergency department. All patients were cured and the median time of hospital stay was 8.0 days (6.0,13.5). In severe-pneumonia group (*N* = 30) the median time of hospital stay was 15.0 days (9.5,23.0) with 27 cases cured and 3 patients left against medical advice.

#### Laboratory and radiological results

Details are shown in Table 2. There was no statistically significant difference in the results of the CBC test (including the level of WBC, neutrophil percentage, hemoglobin, platelets and C-reactive protein) between the pneumonia group and severe pneumonia group. In the liver-kidney function test, ALT (Alanine Aminotransferase in liver function) level in the severe-pneumonia group [34, (16,49)] was higher than that in the pneumonia group [18, (11.5,27.5)] with statistically significant difference (Z = 2.08, *P* = 0.037). There was no statistically significant difference in serum LDH (Lactate dehydrogenase) level between the two groups.

In pneumonia group (*N* = 47), 37 children underwent chest imaging examination (25 chest X-ray and 11 chest computed tomography) and pneumonia was suggested in 36 cases: 15 bilateral involvement (15/36, 41.6%) and 18 unilateral (18/36, 50%). 3 cases could not provide radiological report from the local hospital. In the 36 cases with radiological reported pneumonia 16 had patchy opacities and 7 had consolidation. 13 cases could not provide radiological report from the local hospital.

In severe-pneumonia group (*N* = 30), 28 children underwent chest imaging examination (11 chest x-ray and 17 chest computed tomography) and pulmonary complications were suggested in 11 cases (11/28, 39.2%): 3 pleural effusion, 3 lobar pulmonary consolidation, 3 pulmonary atelectasis and 2 “white lung” like changes (diffuse bilateral pulmonary opacification). In severe-pneumonia group (*N* = 30), complications occurred in 13 cases (13/30, 43.3%) including 7 respiratory failure, 3 atelectasis, 3 pleural effusion and 2 sepsis. No complication occurred in pneumonia or non-pneumonia group.

### Underlying diseases

The profile of underlying diseases in the pneumonia and severe-pneumonia groups was shown in Table 3. 5 children in the pneumonia group (5/47, 10.6%) and 11 children in the severe-pneumonia group (11/30, 36.6%) had underlying diseases, which had a significant difference (*χ*^2^ = 7.571, *P* = 0.006).

**Table 3:** Underlying diseases of the pneumonia and severe-pneumonia group with Chlamydia pneumoniae infection.

Groups	Underlying diseases-no. (%)	Specific diseases	
Pneumonia Group (*N* = 47)	5 (10.6%)	Bronchial asthma (*n* = 1) systemic lupus erythematosus (*n* = 2) epilepsy (*n* = 1) intermediate *α*—thalassemia (*n* = 1)	
Severe-pneumonia Group (*N* = 30)	11 (36.6%)	Bronchial asthma (*n* = 2) Severe malnutrition (*n* = 2) Neonatal hypoxic-ischemic encephalopathy (*n* = 1) Premature in the neonatal period (*n* = 3) Recurrent pneumonia (*n* = 1) Tracheotomy after trauma (*n* = 1) Epilepsy (*n* = 1)	
Comparison between groups		Statistic	*P*-value
		χ^2^ = 7.571	0.006

In the 141 children studied, 18 (18/141, 12.7%) had a history of allergic diseases: 10 had allergic rhinitis, 2 bronchial asthma, 3 cough-variant asthma, 1 eczema, 1 penicillin allergy and 1 had undergone adenoidectomy and tonsillectomy. Among the 18 cases 7 were positive for inhalant allergens and 5 had significantly elevated blood IgE levels.

### Co-infections

Among the 141 children studied, 19 (19/141, 13.4%) had various kind of co-infections. Among children with C. pneumoniae pneumonia, 17 (17/74, 22.9%) had co-infections. Details are shown in Table 4. There was significant difference of the prevalence of co-infections between the pneumonia (3/47, 6.3%) and severe-pneumonia groups (14/30, 46.6%) (*χ*^2^ = 11.14, *P* = 0.001).

**Table 4:** The profile of co-infections in the 77 children with Chlamydia pneumoniae pneumonia^a^.

Groups	Co-infections-no. (%)	Specific pathogens	
Pneumonia Group (*N* = 47)	3 (6.3%)	EB virus (*n* = 1)^b^ Streptococcus pneumoniae (*n* = 1)^c^ Confection of respiratory syncytial virus and Haemophilus influenzae (*n* = 1)^d^^,^c^^	
Severe-pneumonia Group (*N* = 30)	14 (46.6%)	Haemophilus influenzae (*n* = 1)^e^ Adenovirus (*n* = 3)^d^^,^f^^ Co-infection of adenovirus and EB virus (*n* = 1)^d^^,^^g^ Influenza A/B virus (*n* = 2)^h^^,^^f^ Streptococcus pneumoniae (*n* = 2)^e^ Respiratory syncytial virus (*n* = 2)^f^^,^^d^ Parainfluenza virus (*n* = 1)^f^ Stenotrophomonas maltophilia (*n* = 1)^e^ Carbapenem-resistant Klebsiella pneumoniae (*n* = 1)^e^	
Comparison between groups		Statistic	*P*-value
		χ^2^ = 11.14	0.001

a Pathogen detection methods: a. Esophageal leukoplakia was observed under gastroscopy, and fungal infection was suspected.

b The nucleic acid detection of EB virus in blood (fluorescent PCR) was 5.62 × 10^3^ copies/mL.

c Bronchoalveolar lavage fluid culture.

d Seven-in-one detection of respiratory viruses.

e Sputum culture.

f Multiplex nucleic acid detection of respiratory pathogens.

g Serum EBV capsid antigen IgM (ELISA method).

h Rapid detection of influenza A and B by colloidal gold method in nasopharyngeal swabs.

### Multivariate binary logistic regression analysis

Binary logistic regression analysis was performed using the backward likelihood ratio (LR) method to identify independent factors (age, wheezing, ALT level, underlying disease and coinfection) associated with severe C. pneumoniae pneumonia. The final regression model was statistically significant (*χ*^2^ = 27.558, df = 2, *P* = 0.000). The Hosmer-Lemeshow test indicated a good model fit (*χ*^2^ = 2.961, df = 2, *P* = 0.227). The overall correct classification rate of the model was 83.1%. The results showed that wheezing (OR = 15.468, 95% CI: 2.845–84.101, *P* = 0.002) and coinfection (OR = 8.253, 95% CI:1.763–38.630, *P* = 0.007) were independent risk factors for severe C. pneumoniae pneumonia.

### Treatment

In severe-pneumonia group (*N* = 30) 28 cases received macrolides, 1 case tetracyclines and 1 case fluoroquinolones. 8 cases received systemic glucocorticoids and 5 cases immunoglobulin treatment.12 cases received respiratory ventilator support (7 non-invasive and 5 invasive). 6 cases underwent bronchoscopy examination. In pneumonia group (*N* = 47) all cases received macrolides. 3 cases underwent bronchoscopy and 3 cases received systemic glucocorticoids. The average nucleated cell count of the bronchoalveolar lavage fluid was 1,432 × 10^6^/L in pneumonia group (average proportion of neutrophils 41%) and 368 × 10^6^/L in the severe-pneumonia group (average proportion of neutrophils 27%).

## Discussion

Chlamydia pneumoniae is an obligate intracellular pathogen which contains both DNA and RNA nucleic acids. A considerable part of C. pneumonia infection in human is asymptomatic. When it causes respiratory tract diseases it is difficult to distinguish it from the infection of mycoplasma pneumoniae clinically. However, C. pneumoniae infection in the population is not paid so much attention as mycoplasma pneumonia being paid. In addition to acute infection C. pneumoniae infection has also been reported to be related to the occurrence and development of chronic non-infectious diseases such as bronchial asthma, atherosclerosis, Alzheimer's disease and reactive arthritis (17–19). The detection rate of C. pneumoniae in the population with chronic cough is also significantly higher than that in the normal population (20).

Our study showed that the respiratory tract infection of C. pneumoniae in the pediatric population mainly occurs in school-aged children in winter, which was consistent with previous literatures (21–23). The time from onset of disease to test results revealing positive C. pneumoniae antibody was 10.0 days on average. It indicates that a negative antibody test at the beginning of disease cannot completely rule out the possibility of C. pneumoniae infection. Due to different time delays of parents seeking medical treatment the above data may be later than the actual time of antibody presence in the human body.

In our study, the detection methods for serum C. pneumoniae (CP) and mycoplasma pneumoniae (MP) antibody were enzyme-linked immunosorbent assay (ELISA) and particle agglutination method. Notably, in the 141 children with C. pneumoniae infection, 60 cases (60/141, 42.5%) were also positive for MP IgM-antibody or had a MP total antibody titer≥1:1,280. This proportion was much higher than that was reported in the existing literature (24, 25). Whether it is related to the research sample selected in this study or has some association with the detection reagents used in our institution needs to be further verified with larger sample and multi-center data. Serological cross-reactivity between C. pneumoniae and M. pneumoniae may be another possible explanation for this unusually high proportion of coinfection. However, definitive reports on this subject are currently lacking with only a few literatures suggesting sequence homologies between Mycoplasma and Chlamydia spp (26). If the IgM-antibody for CP and MP are both positive, specific nucleic acid detection is required to assist pathogen assertion in greater depth. However, since the treatment for CP and MP infection is similar to a large extent, there is usually no need to make a clear distinction clinically.

In our study, among children with C. pneumoniae pneumonia 22.9% had co-infections. There was significant difference of the prevalence of co-infections between the pneumonia (6.3%) and severe-pneumonia groups (46.6%). Pathogens causing coinfections included viruses (respiratory syncytial virus, adenovirus, influenza A/B virus, parainfluenza virus and EB virus) and bacteria (streptococcus pneumoniae, haemophilus influenzae, stenotrophomonas maltophilia and klebsiella pneumoniae). Except for EB virus, the other pathogens are common pathogens causing respiratory tract infections. Binary logistic regression analysis showed association of coinfections with severe C. pneumoniae pneumonia, which is consistent with the findings reported in other studies on pediatric community-acquired pneumonia (27, 28). However, it cannot be ruled out that certain pathogens may be colonized in the respiratory tract before C. pneumoniae infection such as stenotrophomonas maltophilia and klebsiella pneumoniae. The colonized pathogens may be activated by new-onset infection and aggravate disease severity (29, 30). The shortness of our study is as follows: 1. The 141 children included in this study were all positive for C. pneumoniae detection without a group of healthy children set as control; 2. The study design of this research is a retrospective analysis. A prospective cohort study is needed to further verify the research results; 3. The sample size of this study is relatively small and there may be sampling errors. To obtain more reliable results a larger-sample multi-center study is required.

## Data Availability

The original contributions presented in the study are included in the article/Supplementary Material, further inquiries can be directed to the corresponding author.
